# Editorial: Serving the Public Interest? Micro-Level Perspectives on Antecedents, Motivations, and Values of Pro-Social and Pro-Self Behavior

**DOI:** 10.3389/fpsyg.2021.749277

**Published:** 2021-09-16

**Authors:** Arjen van Witteloostuijn, Hendrik Slabbinck, Richard Walker

**Affiliations:** ^1^School of Business and Economics, Vrije Universiteit Amsterdam, Amsterdam, Netherlands; ^2^Faculty of Economics and Business Administration, Ghent University, Ghent, Belgium; ^3^College of Liberal Arts and Social Sciences, City University of Hong Kong, Hong Kong, SAR China

**Keywords:** public interest, public service motivation (PSM), employer attractiveness, Innovative work behavior, moral values

## Background

For many centuries, one scholar after the other has speculated as to why some societies perform so much better than others. Answers came from many disciplines, such as economics, history, and sociology. In economics, for instance, neo-institutional theory emphasizes the critical role of the formal rules of the game and the informal codes of conduct. Take the well-known work of (e.g., Acemoglu et al., 2005; Robinson and Acemoglu, 2012), arguing that exploitative institutions are the main reason for societal failure, with the elite exploiting the rest of the population for their own benefit. Another well-established and related argument relates to the critical role of trust. Here, economists and sociologists agree that high-trust societies tend to outperform their low-trust counterparts (see, e.g., Zak and Knack, 2001).

What these and many other arguments share is their emphasis on the importance of the production, maintenance, and advancement of high-quality public goods. For societies to prosper, the inhabitants as a collective have to be able to efficiently and effectively coordinate and organize the production of a large collection of public goods, from transportation infrastructures and energy provision to ownership protection and social safety. For that, a large army of people, including many civil servants, are needed to provide the brains and hands required to do so. It is here where psychology comes in, and it is here where the importance of the current Research Topic can be found. After all, for this, society must mobilize many individuals who are intrinsically motivated to invest in the production of public goods—motivated to put the public interest before their self-interest. In public administration, this is referred to as public service motivation.

## This Research Topic

Wang et al. argue that moral theory can contribute to our understanding of public service motivation. In their theoretical contribution, they link psychology's Moral Foundation Theory (MFT) to public administration's concept of Public Service Motivation (PSM) by associating the MFT's five fundamental moral values—Care, Fairness, Authority, Loyalty, and Sanctity—to the individualizing and binding dimensions of PSM. Additionally, they introduce national and organizational culture as boundary conditions.

Li and Xu focus on employee voice as an important condition for public good production. They apply Conservation of Resources Theory (CRT) to develop hypotheses regarding the impact of extraversion and neuroticism on employee voice, adding emotional exhaustion to the mix as a mediator. With survey data from two waves among employees of a Chinese state-owned bank, they provide evidence for a positive indirect effect of extraversion and a negative indirect effect of neuroticism through emotional exhaustion on employee voice.

Slabbinck and Van Witteloostuijn apply psychology's insights regarding the key difference between explicit and implicit motives to the PSM construct. They argue how explicit and implicit need for achievement, affiliation, and power can be expected to be associated differently with different aspects of PSM, focusing on Attraction to Policy-Making (APM), Commitment to the Public Interest (CPI), Compassion (COM), and Self-Sacrifice (SS). They provide evidence for differential associations in two undergraduate student samples from Belgium and the Netherlands, respectively.

Vandenabeele and Jager examine antecedents of the employee attractiveness of public sector organizations, emphasizing the role of PSM as a special case of prosocial motivation in the context of person-organization fit theory. Manipulating the neutral, private, and public sector nature of prospective employer organizations in recruitment messages in a survey vignettes experiment administered among active Dutch job-seekers, they find mixed evidence for PSM's moderating role in explaining a person's preference for public sector employment. To explain the mixed evidence, they mobilize signaling theory.

Bak studies innovative work behavior in a large sample of South Korean local civil servants. Applying insights from organizational support and social exchange theories, the critical role of supervisor feedback is linked to the mediating effect of affective commitment and trust in the supervisor. Both underlying mechanisms are found to significantly mediate the effect of supervisor feedback on innovative work behavior.

Hou et al. investigate participation in charitable activities as an important type of employee volunteering behavior. They present the results from two studies with Chinese samples (MBA students and employees, respectively) into the effect of colleague participation, colleague position, and public cause proximity on employee volunteering intentions. The first study provides evidence for a positive impact of colleague participation and public cause proximity, and the second for a moderating role of power distance vis-à-vis colleagues.

## Future Research

Prosperity in society is closely linked to a well-functioning public sector. For a public sector to function well, public sector organizations must attract employees who are highly motivated to contribute to the public interest. In this Research Topic, Vandenabeele and Jager and Hou et al. examine potential antecedents of serving the public interest, Li and Xu and Bak study drivers of productive and innovative public sector work, and Wang et al. and Slabbinck and Van Witteloostuijn investigate possible determinants of public sector motivation.

Our set of six studies show how many different theories from psychology can be applied to further develop our understanding of issues related to the very important issue of serving the public interest. Of course, this set of six studies cannot but offer a partial understanding of the complex relationships between the antecedents, motivations, and values of public sector attractiveness, public sector productivity, and public sector motivation. We hope this Research Topic increases the interest among psychologists to study these and other questions related to the functioning of the public sector.

## Author Contributions

Guest editing this Research Topic, in all aspects, was a collective effort of all three guest editors. AW took the lead in initiating the Research Topic and writing the editorial. All authors contributed to the article and approved the submitted version.

## Conflict of Interest

The authors declare that the research was conducted in the absence of any commercial or financial relationships that could be construed as a potential conflict of interest.

## Publisher's Note

All claims expressed in this article are solely those of the authors and do not necessarily represent those of their affiliated organizations, or those of the publisher, the editors and the reviewers. Any product that may be evaluated in this article, or claim that may be made by its manufacturer, is not guaranteed or endorsed by the publisher.
